# Flexible Bronchoscopy in a Patient With Pneumomediastinum and Hypoxemic Respiratory Failure: High-Flow Nasal Cannula to the Rescue

**DOI:** 10.7759/cureus.12800

**Published:** 2021-01-20

**Authors:** Sryma PB, Vijay Hadda, Karan Madan, Pawan Tiwari, Anant Mohan

**Affiliations:** 1 Pulmonary Critical Care and Sleep Medicine, All India Institute of Medical Sciences, New Delhi, IND

**Keywords:** pulmonary tuberculosis, pneumomediastinum, high flow nasal cannula, flexible bronchoscopy, ards, acute respiratory distress syndrome

## Abstract

Flexible bronchoscopy with bronchoalveolar lavage carries a significant risk of hypoxia in patients with acute hypoxemic respiratory failure. Noninvasive positive pressure ventilation and high-flow nasal cannula are the most commonly used modalities for reducing procedure-related hypoxemia in such patients. There is no guideline on how to safely perform a bronchoscopy in patients with spontaneous pneumomediastinum and hypoxemic respiratory failure. Here we describe a case of bilateral necrotizing pneumonia, spontaneous pneumomediastinum, and moderate acute respiratory distress syndrome who required diagnostic flexible bronchoscopy.

## Introduction

Fiberoptic bronchoscopy with bronchoalveolar lavage (BAL) holds significant risks of deoxygenation. Bronchoscopy in the intensive care unit is important in the setting of pneumonia not responding to usual treatment or alternate diagnosis being considered. Most guidelines recommend the use of noninvasive ventilation (NIV) for performing flexible bronchoscopy in hypoxemic patients especially in mild-to-moderate acute respiratory distress syndrome (ARDS) [1]. Use of noninvasive positive pressure ventilation (NPPV) reduces the de-saturation associated with the procedure, hence making it safer. Use of NPPV in pneumothorax is a controversial issue, and inserting an intercostal drainage tube is recommended before using NPPV in critical care in such patients [2]. Fatal pneumomediastinum has also been reported with NPPV use [3]. We present the case of a 32-year-old man with bilateral pneumonia with pneumomediastinum who required flexible bronchoscopy for a definitive diagnosis, wherein a high-flow nasal cannula (HFNC) could be successfully utilized for periprocedural oxygenation, to provide definitive diagnosis and prevent the risk of invasive mechanical ventilation.

## Case presentation

**Figure 1:**
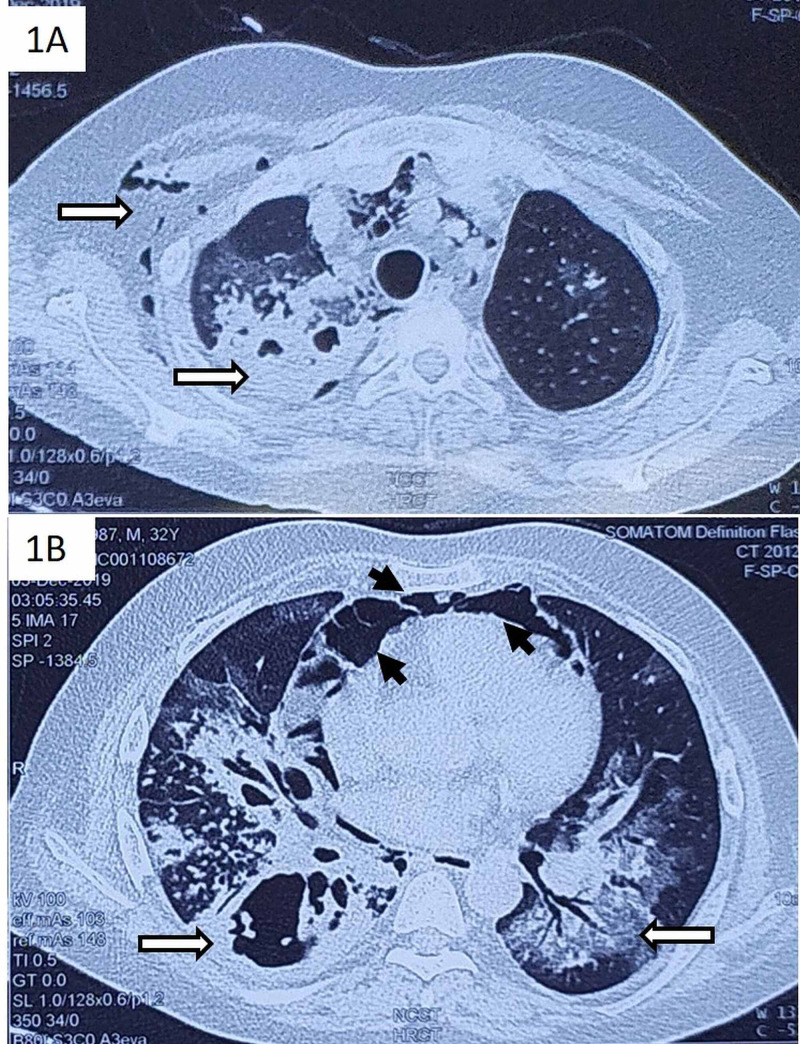
(A, B) High-resolution CT of the chest of the patient showing subcutaneous emphysema on the right side, along with right upper lobe thick-walled cavitation with consolidation (horizontal arrows). Right lower lobe apical segment cavity with bilateral lower lobe consolidation (horizontal arrows) and pneumomediastinum (arrowheads) is also seen.

A 32-year-old Asian male presented with fever, dyspnea, dry cough, weight loss, and loss of appetite for 45 days. Dyspnea progressed from modified Medical Research Council (mMRC) grade 1 to 4 over one week. There was no history of chest pain, hemoptysis, pedal edema, orthopnea, or wheezing. Personal history was significant for uncontrolled diabetes mellitus with a recent glycosylated hemoglobin of 8.9%. There was no history of smoking, alcohol intake, pets at home, or occupational exposure, though he resided in a tuberculosis endemic region. He presented with type 1 respiratory failure with saturation of 89% on supplemental oxygen by face mask at the rate of 15 liters per minute. He had tachycardia (heart rate of 120 beats per minute), tachypnea (resting respiratory rate of 30 breaths per minute), normal blood pressure (110/70 mm Hg). Physical examination revealed bilateral crepitations along with subcutaneous emphysema on the right side of the chest and neck. Investigations showed a total leukocyte count of 8600/mm3, hemoglobin of 11.2 grams per deciliter, and platelet count of 206,000 per mm3. Arterial blood gas analysis showed hypoxemia with respiratory alkalosis, and partial pressure of oxygen in arterial blood was 62 mmHg with a ratio of arterial oxygen partial pressure to fractional inspired oxygen (PaO2/ FiO2) of 155. Chest X-ray showed bilateral consolidation with cavitation. Differential diagnosis at this stage included acute bacterial pneumonia due to gram-negative organisms or staphylococcus, tuberculosis, or fungal pneumonia. Empiric broad-spectrum antibiotics including piperacillin-tazobactam and teicoplanin were started. He could not produce sputum for analysis. High-resolution CT of the chest showed right-sided multifocal consolidation with cavitation in the right upper lobe and bilateral lower lobes, along with pneumomediastinum and subcutaneous emphysema on the right side (Figure 1).

**Figure 2:**
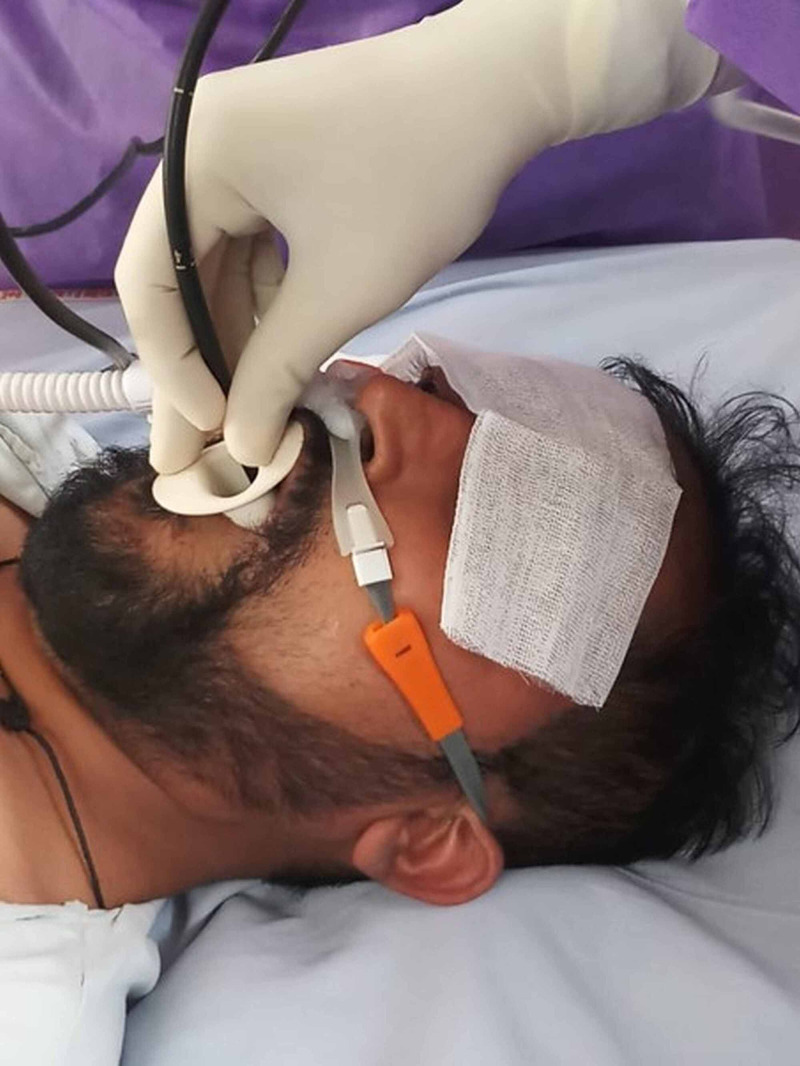
Set up showing a high-flow nasal cannula in situ with a flexible bronchoscope being inserted via the oral route

Blood cultures were sterile. Because of persistent fever, no clinical improvement after 48 hours of antibiotics, and no microbiologic diagnosis, it was decided to proceed with bronchoscopy for microbiologic diagnosis. However, this entailed a risk of worsening hypoxemic respiratory failure and pneumomediastinum. Therefore, it was decided to utilize HFNC for periprocedural oxygenation. Preoxygenation was performed with HFNC of medium adult size as interface with a flow rate of 60 L/min, heated humidification (at 37 degree centigrade), and FiO2 of 1.0 for 5 minutes. The setup is illustrated in Figure 2.

Preprocedure saturation was 99%. Flexible bronchoscopy was performed via the oral route after local anesthesia and conscious sedation with fentanyl. BAL was performed from the right lower lobe apical segment. Saturation remained at 99% for the entire procedure duration. The patient tolerated the procedure well. Postprocedure HFNC was continued for 24 hours. BAL fluid analysis showed acid-fast bacilli positivity, with positive cartridge-based nucleic acid amplification test (CBNAAT) for tuberculosis; BAL bacterial and fungal cultures were negative. The patient was initiated on a four-drug antitubercular therapy (ATT), i.e., rifampicin, isoniazid, ethambutol, and pyrazinamide, according to body weight. He improved with treatment and was discharged home on ATT after two weeks of hospital stay.

## Discussion

Fiberoptic bronchoscopy with BAL carries the risk of worsening hypoxemia, especially in ARDS. The modalities to reduce this risk include oxygen by nasal cannula, NPPV, and HFNC for oxygen delivery. However, NPPV remains a time-consuming and very demanding technique. Patient intolerance to the interface and difficult access of the bronchoscope to the nares due to the facemask are other problems of use of NPPV. HFNC by comparison is easy to use.

A randomized trial to evaluate the modalities of oxygenation during flexible bronchoscopy in acute hypoxemic failure found that application of NIV was superior to HFNC with regard to oxygenation before, during, and after bronchoscopy in patients with moderate-to-severe hypoxemia [4]. However, our situation was complicated by the coexistent pneumomediastinum and subcutaneous emphysema, which may increase with positive pressure ventilation.

There is also a concern that open mouth during the procedure may reduce efficacy of HFNC [5]. The nasal route of flexible bronchoscopy has been tried along with HFNC to prevent this in prospective observational studies [6]. However, this was not clinically significant as our patient had no de-saturation during the entire procedure. HFNC has also been proven effective in retrospective studies on flexible bronchoscopy [7,8].

Spontaneous pneumomediastinum is known to occur due to cavitary tuberculosis, pneumonia, and pneumocystis infections [9-11]. There is no literature on the safe method of performing a flexible bronchoscopy in patients with spontaneous pneumomediastinum. NPPV poses a risk of increasing the mediastinal emphysema. HFNC offers a simpler and probably a safer alternative to NPPV during flexible bronchoscopy in patients with hypoxemic respiratory failure complicated by pneumomediastinum.

## Conclusions

Pneumomediastinum and ARDS pose unique diagnostic challenges, especially in non-intubated patients. NPPV, a commonly utilized oxygenation technique, poses a risk of worsening pneumomediastinum in this scenario. We could successfully use HFNC for periprocedural oxygenation in this case. This modality needs to be further studied for flexible bronchoscopy in patients with hypoxemic respiratory failure.
